# Genome‐Wide Pleiotropy Analysis of Longitudinal Blood Pressure and Harmonized Cognitive Performance Measures

**DOI:** 10.1002/alz70855_103491

**Published:** 2025-12-23

**Authors:** Moonil Kang, Ting Fang Alvin Ang, Sherral A. Devine, Richard Sherva, Shubhabrata Mukherjee, Emily H. Trittschuh, Laura E. Gibbons, Phoebe Scollard, Michael L. Lee, Seo‐Eun Choi, Brandon Klinedinst, Connie Nakano, Logan Dumitrescu, Timothy J. Hohman, Michael L. Cuccaro, Andrew J. Saykin, Walter W. Kukull, David A. A. Bennett, Li‐San Wang, Richard Mayeux, Jonathan L Haines, Margaret Pericak‐Vance, Gerald D. Schellenberg, Paul K Crane, Rhoda Au, Kathryn L. Lunetta, Jesse Mez, Lindsay A. Farrer

**Affiliations:** ^1^ Department of Medicine (Biomedical Genetics), Boston University Chobanian & Avedisian School of Medicine, Boston, MA, USA; ^2^ Framingham Heart Study, Framingham, MA, USA; ^3^ Slone Epidemiology Center, Boston University Chobanian & Avedisian School of Medicine, Boston, MA, USA; ^4^ Department of Anatomy & Neurobiology, Boston University Chobanian & Avedisian School of Medicine, Boston, MA, USA; ^5^ Biomedical Genetics, Department of Medicine, Boston University Medical School, Boston, MA, USA; ^6^ Department of Medicine, University of Washington, Seattle, WA, USA; ^7^ Geriatric Research, Education, and Clinical Center, Veterans Affairs Puget Sound Health Care System, Seattle, WA, USA; ^8^ Department of Psychiatry and Behavioral Sciences, University of Washington School of Medicine, Seattle, WA, USA; ^9^ Department of Medicine, University of Washington School of Medicine, Seattle, WA, USA; ^10^ Vanderbilt Memory and Alzheimer's Center, Vanderbilt University Medical Center, Nashville, TN, USA; ^11^ Vanderbilt Genetics Institute, Vanderbilt University Medical Center, Nashville, TN, USA; ^12^ Vanderbilt Memory & Alzheimer's Center, Vanderbilt University Medical Center, Nashville, TN, USA; ^13^ John P. Hussman Institute for Human Genomics, University of Miami Miller School of Medicine, Miami, FL, USA; ^14^ Department of Medical and Molecular Genetics, Indiana University School of Medicine, Indianapolis, IN, USA; ^15^ Indiana Alzheimer's Disease Research Center, Indiana University School of Medicine, Indianapolis, IN, USA; ^16^ Department of Radiology and Imaging Sciences, Indiana University School of Medicine, Indianapolis, IN, USA; ^17^ Department of Epidemiology, University of Washington School of Public Health, Seattle, WA, USA; ^18^ Rush Alzheimer's Disease Center, Chicago, IL, USA; ^19^ Department of Pathology and Laboratory Medicine, University of Pennsylvania Perelman School of Medicine, Philadelphia, PA, USA; ^20^ Department of Neurology and the Taub Institute for the Study of Alzheimer's Disease and the Aging Brain, Columbia University Irving Medical Center, New York, NY, USA; ^21^ Cleveland Institute for Computational Biology, Department of Population & Quantitative Health Sciences, School of Medicine, Case Western Reserve University, Cleveland, OH, USA; ^22^ University of Washington School of Medicine, Seattle, WA, USA; ^23^ Alzheimer's Disease Research Center, Boston University Chobanian & Avedisian School of Medicine, Boston, MA, USA, Boston, MA, USA; ^24^ Boston University Chobanian & Avedisian School of Medicine, Boston, MA, USA; ^25^ Department of Epidemiology, Boston University School of Public Health, Boston, MA, USA; ^26^ Department of Biostatistics, Boston University School of Public Health, Boston, MA, USA; ^27^ Department of Neurology and Ophthalmology, Boston University Chobanian & Avedisian School of Medicine, Boston, MA, USA; ^28^ Department of Neurology, Boston University Chobanian & Avedisian School of Medicine, Boston, MA, USA.

## Abstract

**Background:**

Genome‐wide association studies (GWAS) have identified over 1,000 blood pressure (BP) loci and over 80 Alzheimer's disease (AD) loci. Considering BP is an AD risk factor, identifying pleiotropy in BP and cognitive performance may indicate mechanistic links between BP and AD.

**Method:**

GWAS for pleiotropy in four BP variables—systolic (SBP), diastolic (DBP), mean arterial (MAP), pulse pressure (PP)—and co‐calibrated scores for cognitive domains (executive function, language, memory) were performed using generalized linear mixed models and 116,075 longitudinal measures from 25,726 participants of clinic‐based and prospective cohorts. Pleiotropy GWAS was conducted using PLACO to estimate SNP's main effect, SNP×age interaction, and their joint effect on pleiotropy. Effects of genome‐wide significant (GWS) pleiotropic SNPs on cognition as direct or mediated through BP were evaluated using Mendelian randomization. Potential contribution of genes in top‐ranked pleiotropic loci to cognitive resilience was assessed by comparing their expression in brain tissue from pathologically confirmed AD cases with and without clinical symptoms.

**Result:**

Pleiotropy GWAS identified GWS associations with *APOE* and 11 novel loci. GWS pleiotropy was identified for SBP and language with SNPs in *JPH2* (*P*_Joint_=6.09×10^‐9^) and *GATA3* (*P*_G×Age_=1.42×10^‐8^) in the total sample and with *RTN4* (*P*_G×Age_=1.49×10^‐8^) in prospective cohorts. GWS pleiotropy was observed for PP and language with *ADAMTS3* (*P*_G_=2.37×10^‐8^) in clinic‐based cohorts. *ULK2* was pleiotropic for DBP and executive function (*P*_Joint_=2.85×10^‐8^) in prospective cohorts. In the total sample, *PAX2* (*P*_G×Age_=4.22×10^‐8^) and *LOC105371656* (*P*_G×Age_=1.75×10^‐8^) had GWS pleiotropy for MAP paired with executive function and language, respectively, and *SUFU* was pleiotropic for DBP and language (*P*_G_=2.10×10^‐8^). *LINC02946* was associated with memory paired with SBP (*P*_G×Age_=3.47×10^‐8^) in clinic‐based cohorts. GWS pleiotropy was found in prospective cohorts for PP and memory with *SORBS2* (*P*_G_=2.33×10^‐8^) and for DBP and memory with *LOC100128993* (*P*_G×Age_=2.81×10^‐8^). Five of the GWS pleiotropic loci influence cognition directly. Genes at six pleiotropic loci were differentially expressed between pathologically confirmed AD cases with and without clinical symptoms.

**Conclusion:**

Our results provide insight into the underlying mechanisms of BP and AD. Ongoing efforts to harmonize BP and cognitive measures across several cohorts will improve the power of discovering, replicating, and generalizing novel associations with pleiotropic loci.

